# Larger quality-of-life gains with an asthma support app: a randomised controlled trial

**DOI:** 10.1183/23120541.00361-2025

**Published:** 2025-11-10

**Authors:** Arnaud Bourdin, Serena Casanova, Engi Ahmed, Benoit Brouard, Adrien Boher, Isabelle Vachier, Carey Suehs, Flore Pujot, Erika Nogue, David Galeazzi, Fanny Cardon, Mathilde Volpato, Laurence Halimi, Sarah Skinner, Nicolas Molinari

**Affiliations:** 1 Department of Respiratory Medicine, CHU Montpellier, University of Montpellier; 2 PhyMedExp, INSERM, CNRS, Montpellier, France; 3 Respiratory Medicine, Department of Translational Medicine, University of Ferrara, Ferrara, Italy; 4 WeFight, Montpellier, France; 5 Médecine Biologie Méditerranée, Montpellier, France; 6 Department of Research and Informatics, University Health, San Antonio, TX, USA; 7 Service d'Information Médicale, Épidémiologie et Données de Santé, CHU Montpellier, Montpellier, France; 8 Direction of Research and Innovation, CHU Montpellier, Montpellier, France; 9 IDESP, INSERM, PreMEdical INRIA, Univ Montpellier, CHU Montpellier, Montpellier, France

## Abstract

**Aim:**

Smartphone applications present an opportunity to offer timely and personalised care to asthma patients. The aim of the study was to determine whether a Chatbot-based application for asthma patients providing educational content and direct access to pulmonology staff improves quality of life, compared with standard therapeutic education.

**Methods:**

This prospective randomised controlled trial (1:1) occurred from 24 May 2022 to 27 October 2023. Patients in the Chatbot group had access to a Chatbot-based, educational smartphone application incorporating Global Initiative for Asthma (GINA) recommendations, and an algorithm validated by experts, including a member of the GINA scientific committee. By interacting with the Chatbot, patients could trigger a mechanism that directly alerted the pulmonology team when patient follow-up was required. The controls (Standard group) partook in standard therapeutic asthma education training. The primary outcome was change in Asthma Quality of Life Questionnaire (AQLQ) scores from baseline to 6 months.

**Results:**

48 of 69 (69.57%) of patients (mean±sd age, 48.43±16.38 years) were female. 29 of 34 (85.3%) in the Chatbot and 29 of 35 (82.9%) in the Standard group had severe asthma (GINA 4–5). Baseline AQLQ, asthma control (ACQ5) and spirometry metrics did not differ between groups. After adjustment, multiple regression analysis indicated that improvements in AQLQ scores were greater in the Chatbot group than in the Standard group (standardised β=0.279, 95% CI 0.004–0.555; p=0.047). Changes in ACQ5 and spirometry metrics did not differ between groups.

**Conclusions:**

Use of the Chatbot-guided application for asthma education and support was associated with greater improvements in quality of life than standard therapeutic education.

## Shareable abstract

**A Chatbot-guided application provided educational content incorporating GINA guidelines and direct contact with pulmonology specialists. Compared with standard therapeutic education, its use was associated with improvements in QoL.** https://bit.ly/4fzXWu8

## Introduction

Despite continuing advancements in asthma diagnosis and treatment, asthma remains a substantial burden for patients and healthcare systems [1]. An estimated 45% of European patients have unacceptable levels of asthma control [2], which increases the risk of exacerbations [3, 4]. As in many chronic diseases, patient education is key to asthma management. Increased asthma knowledge correlates with less rescue medicine use and fewer urgent consults [5]. Educational training programs for asthma patients that are guided by healthcare professionals, and include symptom monitoring, responding to worsening symptoms, and reviewing skills, such as inhaler technique, can effectively reduce asthma morbidity and mortality, and improve quality of life, in both adults and children [6].

Over the past several decades, the use of digital health technologies to improve care delivery and patient education has steadily grown [7, 8]. Digital health interventions and remote monitoring programs can improve medication adherence and quality-of-life outcomes for asthma patients [9, 10]. The extensive adoption of smartphone applications has provided a new platform to provide accessible, low-cost support to patients outside the walls of healthcare facilities. This is particularly relevant in contexts where patients have limited access to healthcare systems, hesitate to seek treatment or underuse their action plans.

To date, the majority of asthma education apps have been created for children [10]. Applications for adults have focused on symptom monitoring and medication adherence, and their efficacy remains inconsistent [9]. However, some asthma applications have shown encouraging results related to improved asthma control, asthma knowledge and quality of life, as well as reduced asthma-related emergency department visits and rescue medication utilisation [11–15]. Others have demonstrated no influence on hospitalisations, adherence, symptoms or quality of life [14, 15]. Furthermore, a systematic evaluation of asthma application content revealed that 39% of applications recommended self-care procedures not supported by evidence, and only 17% provided asthma management and inhaler technique information consistent with guidelines [16]. A second study identified more than 500 asthma-related applications; however, very few of these were evaluated scientifically [17]. The Global Strategy for Asthma Management and Prevention (Global Initiative for Asthma (GINA)) echoed concerns about the quality of asthma applications and underlined the need for high-quality studies to evaluate the effectiveness of applications [6].

Based on previous experience, we felt that three key features of an application would help overcome existing barriers; the application 1) should remain continuously accessible to patients for self-directed use; 2) be designed and reviewed by asthma experts, in line with current GINA care guidelines; and 3) establish a connection with the healthcare team enabling contact when necessary. In this context, the primary objective of the present study was to evaluate the effect of an evidence-based Chatbot-guided application designed for asthma education and support on adults’ Asthma Quality of Life Questionnaire (AQLQ) scores, compared with a standard asthma training program.

## Methods

### Study design and participants

This prospective, randomised, controlled, pilot trial took place at the University Hospital of Montpellier, Montpellier, France. The study was approved by a randomly chosen independent ethics committee (CPP, Ile-de-France VII, 01/20/2022, 21.03617.000059: NCT05248126) [18].

Study presentation and subject recruitment occurred during patients’ routine visits to the University Hospital of Montpellier. Adult patients with a physician-confirmed diagnosis of asthma were eligible to participate. Individuals were excluded if they were not beneficiaries of the French single-payer national medical insurance system, belonged to protected populations according to French Public Health Code L1121-6,8 (*e.g.* were pregnant, parturient, lactating, prisoners or unable to consent), were unable to comply with trial procedures/visits, could experience interference from other studies, previously participated in the study, had already used an asthma-related smartphone application or therapeutic education program or did not give consent. No patients began the intervention and initiated with biologics at the same time.

In accordance with the single-Zelen design, all participating patients gave initial broad consent to participate in the standard patient education program prior to randomisation, and before receiving any information about the study intervention (Consent 1). The patients were then enrolled using an electronic case report form (eCRF) specifically designed for the study. Next, baseline data were collected, the AQLQ and Asthma Control Questionnaire (ACQ-5) were administered, and spirometry was performed according to the American Thoracic Society/European Respiratory Society guidelines [19].

Following baseline data collection, and without the patients’ knowledge, the study team used the eCRFs to perform randomisation (ENNOVCLINICAL), with stratification according to the month of enrolment and asthma severity. A target sample size of 30 patients per arm was established *a priori* as sufficient for demonstrating a significant difference of 1 point (sd of 1.5 points) between the two groups for the primary outcome (AQLQ), with a 1:1 sampling ratio, a type 1 error set at 5% and power at 84%. The difference of 1 point for the AQLQ was chosen because it represents two times the minimal clinically important difference for asthma end-points. Patients randomised to the experimental group were given the opportunity to participate in a novel, Chatbot-guided, smartphone application-based education program. Patients who accepted completed a second round of informed consent (Consent 2), whereas those who refused were excluded from the study. Patients in the Standard group participated in the standard patient education and were not given any information about the Chatbot-guided program. At the end of the inclusion visit, all patients received a health resource use diary. Telephone calls to promote program adherence and collect data related to exacerbations, health resource utilisation and medications occurred at weeks 4, 8, 12, 16 and 20. The end-of-study visit occurred during a routine consultation 6 months after inclusion. The AQLQ and ACQ-5 questionnaires were administered, and spirometry measures was performed again. The patients’ health resource diaries were retrieved, as were data concerning the number of telephone calls and emails addressed to patients, and the frequency and volume of patients’ Chatbot use.

#### Interventions

Inclusion visits occurred face-to-face for patients in both groups. During this initial visit, the nurse and patient discussed questions and priorities related to asthma management, and ultimately created a personalised action plan. Personalised treatment goals were related to seven domains, based on GINA guidelines for asthma self-management education and skills training [6]: treatment management (managing medications and treatment methods during maintenance phases, exacerbations and emergencies), symptom perception and tracking, environmental management (tracking environmental stimuli, allergen avoidance, vaccinations), smoking cessation, physical activity, psychosocial support and disease knowledge (supplementary table S1).

##### Experimental intervention: clinically integrated, Chatbot-guided, self-management educational support tool

The multidisciplinary team (pulmonologist, asthma nurse, physiologist, psychologist, smoking-cessation nurse and social worker) in charge of classic therapeutic training at the University Hospital of Montpellier developed the application in collaboration with WeFight, a private company that develops applications to support patients with chronic diseases. The Chatbot was designed to be clinically integrated and to respond to patient queries (supplementary figure S1) pertaining to the seven domains described by the GINA guidelines (supplementary table S1).

The Chatbot application automatically sent the patients’ data to a local Electronic Health Record platform (MedVik) that was created for the study. The application included programming that deployed alerts to designated medical staff *via* the MedVik platform, based on patients’ input into the application. Depending on the words used by the patient, or their input frequency, the algorithm classified alerts as green, orange or red, which were displayed on a specific dashboard reviewed by nursing staff each morning (supplementary figures S2 and S3). An orange-level alert triggered an email contact to the patient to determine whether the patient's asthma symptoms were worsening and to help manage a potential episode. A red-level alert resulted in a phone call and potentially an unscheduled visit to the pulmonology department. Ten nurses received 8 h of specific training (two 4-h sessions during normal work hours) so that they could competently use the application, check the dashboard and respond to alerts.

The initial version of the application was pilot tested by the study investigators and several patients with asthma. The feedback provided during the pilot testing was integrated into the version of the application used in the intervention. The Chatbot was designed to be usable without any specific training.

The nurses helped each subject download the Chatbot application to their smartphone during the inclusion visit. Based on each patient's personalised action plan, the nurses helped the patient select three of the seven domains (supplementary table S1) most pertinent to their treatment goals. The nurse then personalised the Chatbot's settings for each patient to provide information related to these three domains. The patients were only prompted to use the application if they had not interacted with it for 3 weeks in a row. In these cases, the application sent a question related to the three domains initially selected by the patients using a warm and friendly tone.

##### The comparator intervention: standard therapeutic training for asthma patients

The Standard group participated in the usual therapeutic educational training program currently used in the General Pulmonology Unit at the University Hospital of Montpellier, and approved by the French Regional Health Authority. After establishing the patient's personalised action plan during the inclusion visit, further therapeutic training sessions occurred as often as necessary, as determined by the patient–caregiver dyad and associated goals. Email and phone numbers were provided to patients so they could contact the department in case of asthma exacerbations.

During subsequent training sessions, the patient's treatment was discussed, with emphasis on treatment goals and treatment adherence. The nurse also presented different types of inhalers and coached the patient on proper inhaler technique. When appropriate, other therapeutic areas, including smoking cessation, trigger and allergen avoidance, lifestyle and occupational adaptation and physiotherapy were discussed.

### Outcomes

The primary outcome of the study was change in total AQLQ score, which reflects quality of life between the inclusion visit and 6 months. We chose to focus on this measure because the overarching goal of self-management education is to enhance quality of life by improving symptom control and overall asthma outcomes. The AQLQ evaluates both physical and emotional aspects of the disease, is easy to use and correlates with asthma control [20]. Secondary outcomes were the number of hospitalisations, change in spirometry measures (forced expiratory volume in 1 s (FEV_1_) and forced vital capacity (FVC)) and changes in inhaled corticosteroid dose (µg·day^−1^), ACQ5 score and EuroQoL Five-Domain Five-level questionnaire score.

### Statistical methods

Descriptive statistics were summarised as numbers (percentages) for qualitative variables, and as mean±sd or medians with the first and third quartiles (25% to 75%) for quantitative variables. Patients’ baseline demographic and clinical characteristics were compared between the Standard and Chatbot groups using Pearson's chi-squared tests or Fisher's exact test for qualitative data, and two-sample t-tests or Wilcoxon rank-sum tests for quantitative data.

The primary outcome was compared between the Standard and Chatbot groups using a Wilcoxon rank-sum test. A Forest plot was created to illustrate the differences in change in AQLQ between the Standard and Chatbot groups with their 95% confidence intervals in different subgroups for age, GINA step, sex, smoking habits, obesity and eosinophils. Subjects with a change in total AQLQ>0.5 [20] were classified as “responders” and the percentage of “responders” in each group was compared using a Pearson's chi-squared test. Simple linear regression was used to model the relationship between the primary outcome (change in AQLQ) and group (Standard *versus* Chatbot groups), and then multiple linear regression was conducted to assess the relationship between the primary outcome and group, with adjustment for asthma severity (according to GINA step, classified into two groups: GINA 1–3 *versus* 4–5) and number of connections (defined as the number of times the patient logged into/opened the Chatbot application on their smartphone between their inclusion in the study and the end date of the study). Raw and standardised regression coefficients were reported with their 95% confidence intervals. The same analyses were conducted using logistic regression to assess the relationship between “responder” status (change in total AQLQ>0.5) and group. Changes in secondary outcomes (number of hospitalisations, FEV_1_, FVC and ACQ5) were compared between groups using Wilcoxon rank-sum tests. For all analyses, a two-sided p-value <0.05 defined significance. Missing data were not imputed. All data analyses were conducted using R v.4.3.1.

## Results

### Participant flow

**FIGURE 1:**
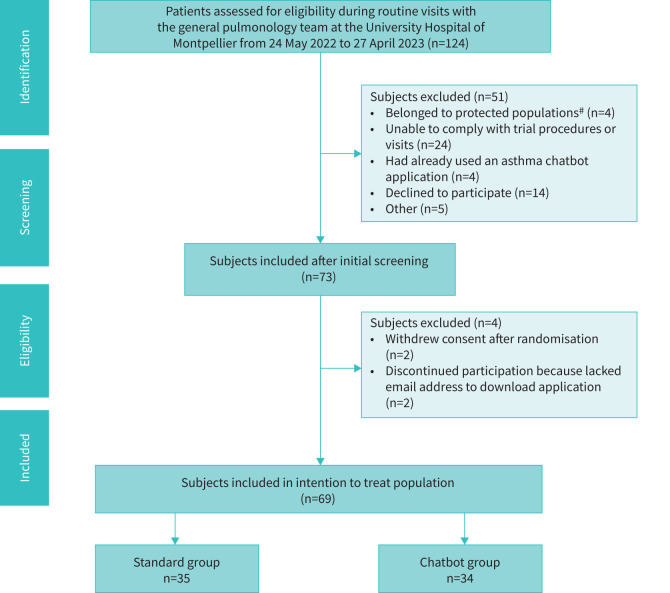
Study flowchart. ^#^: protected populations according to the French Public Health Code Articles L 1121-6,8 (*e.g.* people who were pregnant, parturient, lactating, prisoners or unable to consent).

Out of 124 patients considered for inclusion, 73 patients were initially included in the study (between 24 May 2022 and 27 October 2023) and 69 subjects were ultimately considered in the intention-to-treat population (details in figure 1).

### Baseline data

Baseline demographic and clinical characteristics of patients in the Standard (n=35) and Chatbot (n=34) groups, and between group comparisons of these variables, are shown in table 1. All subjects were treated with inhaled corticosteroids, the majority received GINA step 4 or 5 treatment, and almost half received biologic therapy at baseline. Overall, five patients initiated treatment with biologics during the intervention period; two in the Standard group and three in the Chatbot group.

**TABLE 1:** Baseline demographic and clinical characteristics of patients in the Standard and Chatbot education training groups

	Standard, n=35	Chatbot, n=34	p-value
**Female**	27 (77.14)	21 (61.76)	0.165^¶^
**Age, years**	52.03±17.20	44.74±14.84	0.064^+^
**GINA class 4 or 5**	29 (82.86)	29 (85.29)	0.782^¶^
**Asthma Quality of Life Questionnaire**	4.26±1.41	4.39±1.35	0.720^+^
**Asthma Control Questionnaire 5**	2.38±1.26	2.12±1.30	0.417^+^
**EuroQoL 5-Domain 5-Level Questionnaire**	0.81±0.25	0.82±0.24	0.542^##^
** **Missing	1	1	
**Currently employed**	15 (53.57)	13 (56.52)	0.833^¶^
** **Missing	7	11	
**Tobacco use**			0.354^§^
** **Nonsmoker	22 (66.67)	18 (56.25)	
** **Former smoker	9 (27.27)	8 (25.00)	
** **Current smoker	2 (6.06)	6 (18.75)	
** **Missing	2	2	
**BMI, kg·m^−2^**	27.64±5.43	26.97±4.78	0.594^+^
** **Missing	2	0	
**≥1 exacerbation in the past 12 months**	31 (88.57)	32 (94.12)	0.673^ƒ^
**Number of exacerbations in the past 12 months**	3.00 (2.00–5.00)	4.00 (2.00–6.50)	0.276^##^
** **Missing	4	2	
**≥1 Positive skin-prick test^¶¶^**	19 (54.29)	23 (67.65)	0.256^¶^
**Chronic rhinosinusitis**	27 (79.41)	24 (72.73)	0.521^¶^
** **Missing	1	1	
**Aspirin intolerance**	4 (11.76)	5 (15.15)	0.734^ƒ^
** **Missing	1	1	
**Atopic dermatitis**	8 (23.53)	9 (27.27)	0.725^¶^
** **Missing	1	1	
**Obesity**	12 (35.29)	3 (9.09)	**0.010^¶^**
** **Missing	1	1	
**Anxiety/depression**	13 (40.63)	15 (45.45)	0.694^¶^
** **Missing	3	1	
**Eosinophils, 10^6^·L^−1^**	315.50 (202.50–635.00)	322.00 (142.50–740.00)	0.801^##^
** **Missing	1	0	
**Forced vital capacity**	94.71±17.50	96.12±17.22	0.736^+^
**Forced expiratory volume in 1** **s**	82.00 (66.78–95.50)	86.00 (75.50–98.50)	0.296^§^
**ICS treatment^#^**			0.348^¶^
** **GINA track 1	29 (82.86)	25 (73.53)	
** **GINA track 2	6 (17.14)	9 (26.47)	
**Levels of daily beclometasone equivalent dose of ICS^++^**			0.229^¶^
** **<500 µg·day^−1^	8 (22.86)	5 (15.15)	
** **500–1000 µg·day^−1^	15 (42.86)	21 (63.64)	
** **>1000 µg·day^−1^	12 (34.29)	7 (21.21)	
**Oral corticosteroids**	4 (11.43)	1 (2.94)	0.356^ƒ^
**Biologic therapy**	15 (42.86)	14 (41.18)	0.888^¶^

Data are presented as n (%), mean±sd or median (interquartile range). GINA: Global Initiative for Asthma; BMI: body mass index; ICS: inhaled corticosteroid; LABA: long-acting β-agonist; LAMA: long-acting muscarinic antagonist; SABA: short-acting β-agonist. **^#^:** GINA track 1, ICS formoterol as a maintenance and reliever therapy; GINA track 2, fixed dose ICS–LABA+SABA as a reliever therapy. 16 patients in the GINA track 1 group and 4 patients in the GINA track 2 group were also treated with LAMAs. ^¶^: Pearson's chi-squared test. ^+^: two-sample t-test. ^§^: Fisher's exact test for count data with simulated p-value (based on 2000 replicates). ^ƒ^: Fisher's exact test. ^##^: Wilcoxon rank-sum test. ^¶¶^: Or at least one specific IgE ≥0.35 kUI·L^−1^ to pneumallergens. ^++^: Dose calculated does not include ICSs taken according to smart strategy (n=68).

### Outcomes

**FIGURE 2:**
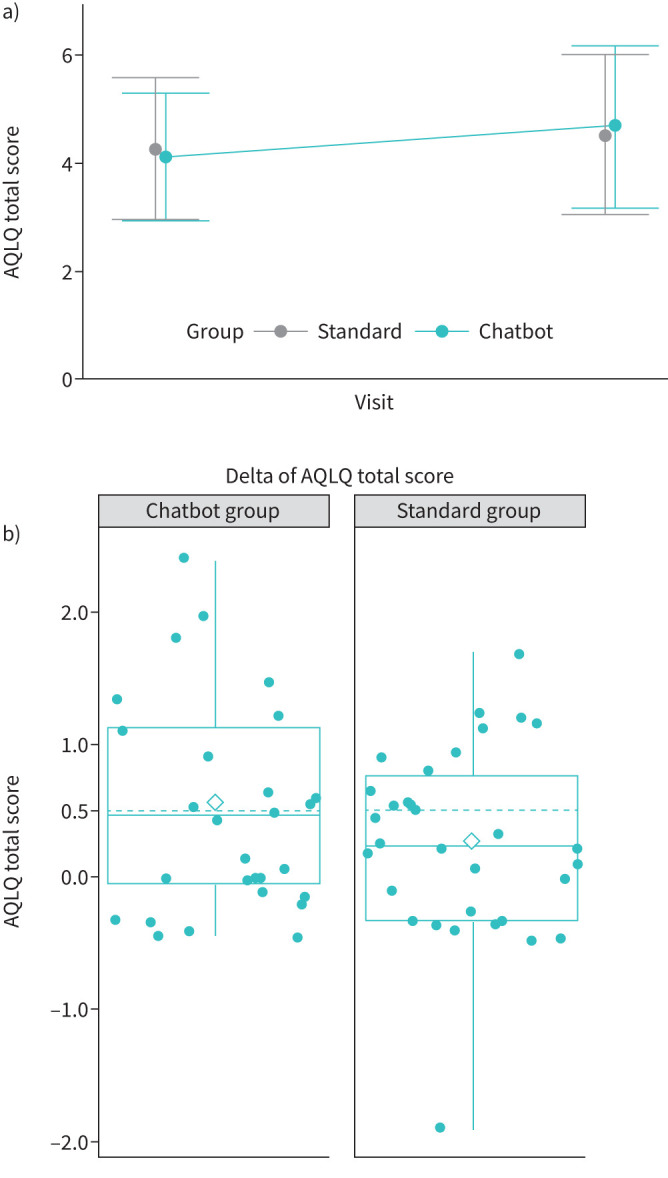
a) Mean±sd Asthma Quality of Life Questionnaire (AQLQ) total scores in the Standard and Chatbot groups at day 0 (the initial visit) and at month 6 (the end-of-study visit). b) Primary outcome, change in AQLQ total score. The white diamond represents the mean, the middle line represents the median and the lower and upper hinges correspond with the first and third quartiles (the 25th and 75th percentiles). The upper whisker extends from the hinge to the largest value no further than 1.5 × interquartile range (IQR) from the hinge. The lower whisker extends from the hinge to the smallest value at most 1.5 × IQR of the hinge.

**FIGURE 3:**
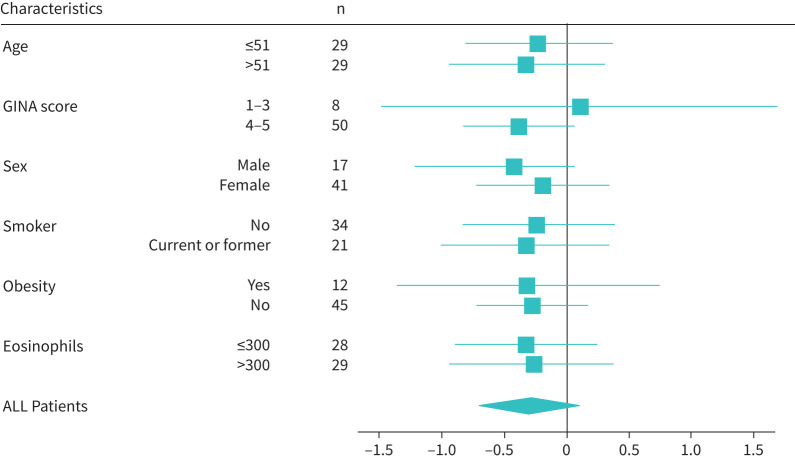
Differences in change in Asthma Quality of Life Questionnaire (AQLQ) between the Standard and Chatbot groups by subpopulation. A Forest plot was created to illustrate the differences in change in AQLQ between the Standard and Chatbot groups (difference=Standard group mean − Chatbot group mean) with their 95% confidence intervals in different subgroups for age, Global Initiative for Asthma (GINA) score, sex, smoking habits, obesity and eosinophils.

Median (interquartile range) change in AQLQ scores between baseline and 6 months was greater in the Chatbot group (0.47 (−0.05–1.13); n=28) than in the Standard group (0.23 (−0.33–0.77); n=30), although the difference did not reach significance (figure 2). supplementary table S2 shows changes in the domains of the AQLQ. In the Chatbot group, 13 (54.17%) subjects were classified as “responders” and 11 (45.83%) were classified as “responders” in the Standard group (p=0.41). Four (11.43%) patients in the Standard group were hospitalised during the intervention, whereas no patients were hospitalised in the Chatbot group. No patients required intensive care during the study period. The forest plot (figure 3) illustrates that no subpopulation of patients responded better to the Chabot-guided training, but improvements in AQLQ scores tended to be larger in the Chatbot group in all subgroups, except the GINA 1–3 severity group. Changes in secondary outcome values from baseline to 6 months are shown in table 2. Subjects in the Chatbot group connected to the Chatbot a mean±sd of 13±28.18 times and a median of 2.5 connections (interquartile range 1.25–6.5) (supplementary figure S4). Data related to the burden of the intervention on the healthcare team are shown in supplementary table S3.

**TABLE 2:** Changes in secondary outcome values from baseline to 6 months

Variable	Standard (n=35)	Chatbot (n=34)	p-value
**Forced expiratory volume in 1** **s**	−4.00 (−7.63–1.00)	2.00 (−4.00–8.00)	0.11^#^
Missing	9	9	
**Forced vital capacity**	−1.00 (−5.00–3.00)	0.00 (−6.00–10.00)	0.30^#^
** **Missing	10	9	
**Annualised exacerbation rates**	−0.18±3.82	−1.79±3.88	0.14^¶^
** **Missing	7	10	
**Asthma Control Questionnaire**	−0.20 (−1.00–0.20)	−0.40 (−0.85–0.05)	0.68^#^
** **Missing	6	6	
**The EuroQoL 5-Domain 5-Level Questionnaire**	0.00 (−0.08–0.03)	0.00 (−0.02–0.03)	0.220^#^
** **Missing	7	6	
**Inhaled corticosteroid dose, µg·day^−1+^**	0.00 (0.00– 0.00)	0.00 (0.00–0.00)	0.990^#^
** **Missing	0	0	

Data are presented as n, mean±sd or median (interquartile range). ^#^: Wilcoxon rank-sum test. ^¶^: two-sample t-test. ^+^: beclometasone equivalent dose.

In the multiple linear regression model, we adjusted for asthma severity (GINA score 1–3 *versus* 4–5 classification) and the number of Chatbot connections. Asthma severity was included to account for baseline differences in disease burden, which may influence quality of life outcomes (figure 3). The number of Chatbot connections was included due to its highly skewed distribution and its potential to reflect varying levels of patient engagement with the intervention. The results of the multiple linear analysis (tables 3 and 4) indicated that “group” was the variable in the model that had the greatest effect on change in AQLQ score. The Chatbot group was associated with greater improvement in total AQLQ score change compared with the Standard group (β_SD_=0.279, 95% CI 0.004–0.555; p=0.047), after adjusting for asthma severity and number of connections to the Chatbot (table 3).

**TABLE 3:** Results (β values) of regression models used to assess the relationship between group (Standard, n=30; Chatbot, n=28) and change in the AQLQ score

Δ AQLQ^#^		β	95% CI	β_SD_	95% CI	p-value
**Simple linear regression model**						
**Group**	Standard	NA	NA			0.163
	Chatbot	0.293	−0.122–0.709	0.186	−0.077–0.449	
**Multiple linear regression model**						
**Group**	Standard	NA	NA			0.047*
	Chatbot	0.442	0.007–0.877	0.279	0.004–0.555	
**GINA score**	4, 5	NA	NA			0.174
	1–3	0.408	−0.186–1.002	0.178	−0.081–0.437	
**Number of connections to Chatbot**		−0.008	−0.018–0.002	−0.231	−0.506–0.043	0.097

AQLQ: Asthma Quality of Life Questionnaire; NA: not applicable; GINA: Global Initiative for Asthma. ^#^: Δ AQLQ: change in total AQLQ score as a continuous variable. *: p<0.05.

**TABLE 4:** Results of regression models (odds ratios) used to assess the relationship between group (Standard, n=30; Chatbot, n=28) and change in the AQLQ score

Δ AQLQ>0.5^#^		OR	95% CI	OR_SD_	95% CI	p-value
**Simple logistic regression model**						
**Group**	Standard					
	Chatbot	1.497	0.525–4.346	1.226	0.723–2.097	0.450
**Multiple logistic regression model**						
**Group**	Standard					0.119
	Chatbot	2.635	0.797–9.282	1.630	0.892–3.074	
**GINA score**	4, 5					0.100
	1–3	3.878	0.775–24.667	1.602	0.915–3.050	
**Number of connections to Chatbot**		0.958	0.885–1.001	0.377	0.064–1.022	0.058

AQLQ: Asthma Quality of Life Questionnaire; GINA: Global Initiative for Asthma. ^#^: Δ AQLQ>0.5: Change in total AQLQ score as a dichotomous variable. Subjects with a change in total AQLQ >0.5 were classified as “responders” and those with change in total AQLQ scores ≤0.5 were considered “nonresponders”.

The results of the multiple logistic regression analysis (table 4) showed that the relationship between group and responder status did not reach significance (OR_SD_=1.630, 95% CI 0.892–3.074; p=0.119). The number of connections tended to be associated with the response status (OR_SD_=0.377, 95% CI 0.064–1.022; p=0.058).

## Discussion

This randomised controlled study examined the effects of a clinically integrated, Chatbot-guided smartphone application, specifically designed for asthmatic adults, on quality of life. Our results indicate that participation in the Chatbot group tended to be associated with greater improvements in quality of life over the 6-month period, compared with Standard therapeutic education. Additionally, no patients in the Chatbot group were hospitalised, whereas 11.43% of patients in the Standard group were hospitalised during the intervention. This finding is meaningful as hospitalisations contribute significantly to the burden of the disease on the patients and the healthcare system.

Our results are in alignment with a recent Cochrane systematic review that reported that digital health interventions could improve asthma patients’ quality of life [9]. This conviction was also underlined in the most recent GINA report [6]. However, results are not unanimous when considering smartphone applications specifically for adults with asthma [11, 12, 14]. For example, Kandola *et al.* [13] reported that using an asthma management application for 8 weeks improved asthma symptom control in adults, but had no impact on users’ quality of life. Additionally, Ryan *et al.* [14] examined a smartphone application that helped asthma patients monitor symptoms, medication use and lung function, provided immediate feedback related to their personalised action plan and alerted asthma nurses when the patients’ health status declined. The authors found that changes in asthma control, healthcare resource utilisation and quality of life did not differ significantly between application users and controls. However, the authors did not conduct any further regression analyses to determine whether the application was associated with changes in quality of life.

Although in-person asthma education training is a recognised care solution that improves asthma knowledge, asthma control and quality of life [21–26], few studies specifically focus on application-based educational training programs for asthmatic adults. A study by Hsia *et al.* [12] showed that using an educational asthma application could improve asthma knowledge and quality of life. However, the smartphone application was only accessible to participants during a single study visit, and the study lacked a control group. More recently, Rudin *et al.* [22, 24] designed a scalable, application-based symptom monitoring intervention for patients with asthma that included access to educational material. As in our study, their application was clinically integrated and enabled patients to contact nursing staff when needed. Although the 12-month intervention resulted in improvements in quality of life, the changes were not clinically significant [24]. Interestingly, certain subgroups of patients, including those 18–44 years old, and those with low activation, low miniAQLQ scores and uncontrolled asthma at baseline showed greater improvements in quality of life relative to the usual care group [24]. Application usage was much higher in their cohort of patients (retention of 78%) compared with ours. This could be because the authors designed the application using a user-centred design process that followed the non-adoption, abandonment, scale-up, spread and sustainability framework. As a result, their application reflected the needs of the patients [22].

In our study, the observed improvement in quality of life in the Chatbot group likely reflects not only the application's educational content but also its integration into clinical care and its support for symptom perception and tracking. Although the application did not offer self-monitoring of physiological measures, it functioned as a self-management support tool by incorporating structured assessments that helped patients monitor their own symptoms, empowering them to make informed decisions about their asthma care. Furthermore, the application was not designed to completely replace interactions with healthcare professionals, but to be integrated into patients’ clinical care pathways. Like in the Standard group, patients in the Chatbot group attended an essential initial in-person visit that enabled them to establish their personalised action plan with the support of a trained nurse. The application also included an algorithm that sent alerts to the care team depending on the patient's input in the app. The Chatbot tool proposes an “on demand” approach to healthcare, which facilitated contact between the patient and the healthcare team before the patient's symptoms deteriorated, and allowed them to consult educational materials when they feel most motivated and engaged. However, if the patient wished to contact the healthcare team directly, they still needed to call or email the clinic. Applications that include an alert system, and a functionality that allows patients to contact the healthcare team directly could present a particular benefit in contexts where access to asthma training and support is limited, either due to time constraints related to patients’ professional or personal responsibilities, busy respiratory clinics or healthcare deserts.

Implementing the Chatbot program required direct interaction with the nurses that was intended to foster engagement in the program. However, this approach did add an extra burden to the nursing staff. Future studies could explore whether automating these processes, for example by incorporating a questionnaire into the application to help patients select three of the seven most pertinent domains, could reduce the burden. The patient could complete the questionnaire at regular intervals, allowing the application to automatically update to patient needs.

Despite the promising aspects of the application, overall engagement with the Chatbot was modest, and the distribution of connections skewed positively, suggesting that while a few patients used the app intensively, many engaged only occasionally. Research on patients’ use of health applications has identified low engagement rates as a barrier to their use [25, 26]. Few patients use health applications on a regular and long-term basis, and concerns around privacy and data management remain [25, 26]. Future studies could explore ways to promote use of application-based educational training among adult asthma patients.

We included the number of Chatbot connections as a covariate in our multiple regression models to control for the varied levels of patient engagement. The trend observed for greater AQLQ scores in patients who used the Chatbot more frequently may reflect the benefits of increased engagement, more consistent exposure to educational content, and timely access to personalised support. Our multiple regression models also adjusted for disease severity, measured using the GINA score. Severity was included to account for baseline differences in disease burden, which may influence the potential for improvement in quality of life.

We chose to evaluate AQLQ, a quality-of-life measure, as our primary outcome because the overarching goal of self-management education is to enhance quality of life by improving symptom control and asthma outcomes. Additionally, improvements in total AQLQ scores are significantly associated with FEV_1_, forced mid-expiratory flow between 25–75% of vital capacity and morning peak expiratory flow [27]. AQLQ score is also negatively correlated with asthma-related emergency department use [28]. In the present study, we did not observe significant improvements in ACQ5 or spirometry measures in the Chatbot group, compared with the Standard group. This could be attributed to the Standard group's participation in the therapeutic training program for asthma patients offered at the University Hospital of Montpellier, which likely helped patients improve or maintain asthma control and lung function better than if they had not received any specialised care. If the Chatbot group was instead compared with a control group of patients followed by a general practitioner, we might have observed differences in asthma control and/or lung function. Notably, the majority of subjects in the study had severe asthma, which may have also influenced the spirometry results.

This randomised controlled study used a single-Zelen design, in which only the patients randomised to receive the smartphone application were made aware of the intervention, preventing individuals in the control arm from experiencing any “resentful demoralisation” [29]. Another strength was that the smartphone application's content was aligned with GINA recommendations. Despite careful planning, the present study does contain several limitations. First, it is not multicentric, thereby limiting the generalisability of our results. Additionally, our hypothesis of superiority, that the application would perform better than standard therapeutic training, may have been too ambitious, possibly resulting in the under-powering of our study. A hypothesis of non-inferiority might have been a more appropriate methodological approach. In retrospect, using a win ratio to consider multiple outcomes (including ACQ5 scores, oral corticosteroid use and lung function) and to capture cumulative rare events (such as hospitalisations, unscheduled healthcare visits and emergency department admissions) could have been another pertinent approach. Our study also mainly included severe asthma patients, who may not be the most relevant population for the application. Patients with less severe asthma and less frequent medical visits may have responded differently. Self-management interventions have been shown to reduce health resource use and improve quality of life in individuals with asthma [30]. As our study was not designed to evaluate health resource use, future studies should specifically evaluate the impact of a similar intervention on this outcome. Another important limitation of this study was the discontinuation of the app following the closure of the private company that developed it. This event underscores a broader challenge in digital health: the risk of care disruption when clinical workflows become dependent on proprietary technologies without long-term sustainability plans. Considering this experience, alternative models such as open-source or publicly supported platforms may offer more resilient and equitable solutions. Finally, although smartphone use is seemingly pervasive, there remain certain individuals who either do not have access to the technology, do not know how to use the technology or are reticent to use applications because of concerns related to sharing of personal data. We did not collect any qualitative data in our study. Doing so could have helped identify mechanisms to explain how participation in the Chatbot arm was associated with improved quality of life, despite a relatively low number of connections to the application.

### Conclusion

Smartphone applications present an accessible solution to provide people with education and skills training to manage their asthma, and to facilitate communication with healthcare professionals. Our results indicate that an application-based self-management education program for adults with asthma could help improve patients’ quality of life. Promoting consistent and persistent use of the application among all patients remains a challenge. Identifying and understanding patients’ motivations for using the application could help improve adherence. Future studies should also evaluate whether application-based self-management education and skills training can reduce healthcare resource use. Exploring how to streamline implementation of similar interventions could help improve their scalability so that the application could benefit more people.

## Data Availability

Individual patient data will not be shared.

## Supplementary material

**Please note:** supplementary material is not edited by the Editorial Office, and is uploaded as it has been supplied by the author. Supplementary material 00361-2025.SUPPLEMENT
