# Supplementary material for: Larger quality-of-life gains with an asthma support app: a randomised controlled trial
Source: ERJ Open Res. 2025 Nov 10;11(6):00361-2025. doi: 10.1183/23120541.00361-2025 (PMC12598602; doi:10.1183/23120541.00361-2025)

SUPPLEMENTARY MATERIALS

TABLES

Supplemental Table 1: Seven domains of focus of therapeutic education for patients with asthma

Seven domains of focus of therapeutic education for patients with asthma, based on GINA Guidelines*
(1) Treatment management (during maintenance phases, exacerbations and emergency situations, and how treatments are taken - particularly inhaler technique)<br>(2) Symptom perception and tracking<br>(3) Environmental management (indoor and professional environments, allergen management, vaccinations)<br>(4) Smoking cessation<br>(5) Physical activity<br>(6) Psychosocial support<br>(7) Disease knowledge
*For participants in the Chatbot group, the nurse helped each patient choose three of the domains most pertinent to their treatment goals. The nurse then personalized the Chatbot’s settings to provide information related to the patient’s three chosen domains.

Supplemental Table 2: Scores for AQLQ domains at day 0, month 6, and the change in each score from day 0 to month 6

AQLQ domains,	**Day 0**			**Month 6**			**Delta (Month 6 Day 0))**		
Median (25%; 75%)	Standard, N = 35	Chatbot, N = 34	p-value	Standard, N = 35	Chatbot, N = 34	p-value	Standard, N = 35	Chatbot, N = 34	p-value
Symptoms	4.00 (3.44 ; 5.11)	4.08 (3.42 ; 5.33)	0.551[2]	4.42 (3.05 ; 5.67)	4.63 (3.77 ; 5.94)	0.348[2]	0.09 (-0.25 ; 0.81)	0.25 (-0.12 ; 1.08)	0.189[2]
Activity limitation	4.05 (3.16 ; 5.39)	4.18 (3.27 ; 5.91)	0.693[1]	4.45 (3.64 ; 5.73)	4.87 (3.71 ; 6.27)	0.489[2]	0.09 (-0.16 ; 0.87)	0.32 (0.16 ; 1.32)	0.150[1]
Emotional function	4.80 (3.25 ; 5.75)	4.40 (3.60 ; 5.60)	0.983[2]	4.60 (3.20 ; 6.10)	4.80 (4.00 ; 6.05)	0.773[1]	0.00 (-0.35 ; 0.95)	0.30 (-0.05 ; 0.80)	0.354[1]
Environmental stimuli	5.00 (3.06 ; 5.75)	4.50 (2.75 ; 5.75)	0.723[2]	4.50 (3.13 ; 6.13)	4.38 (2.88 ; 5.81)	0.606[2]	0.25 (-0.25 ; 0.50)	0.00 (-0.50 ; 1.13)	0.752[2]
Missing	1	1		4	6		5	6	

[1]Wilcoxon rank sum test

[2] Two Sample t-test

Supplemental Table 3: Burden for medical staff and consultations measured from baseline to six months, reported as average number (± SD) per patient

	Standard, N = 35	Chatbot, N = 34	p-value*
Emails to the patient	1.37 (± 2.13)	1.24 (± 1.65)	0.891
Telephone calls to the patient	4.97 (± 1.72)	4.59 (± 1.54)	0.176
Planned medical consultations	0.94 (± 2.24)	0.97 (± 1.34)	0.584
Unplanned medical consultations	1.23 (± 1.54)	0.97 (± 1.57)	0.369

*Wilcoxon rank sum test

FIGURES

Supplemental Figure 1: Screenshots of the Chatbot application interface with prompts and responses

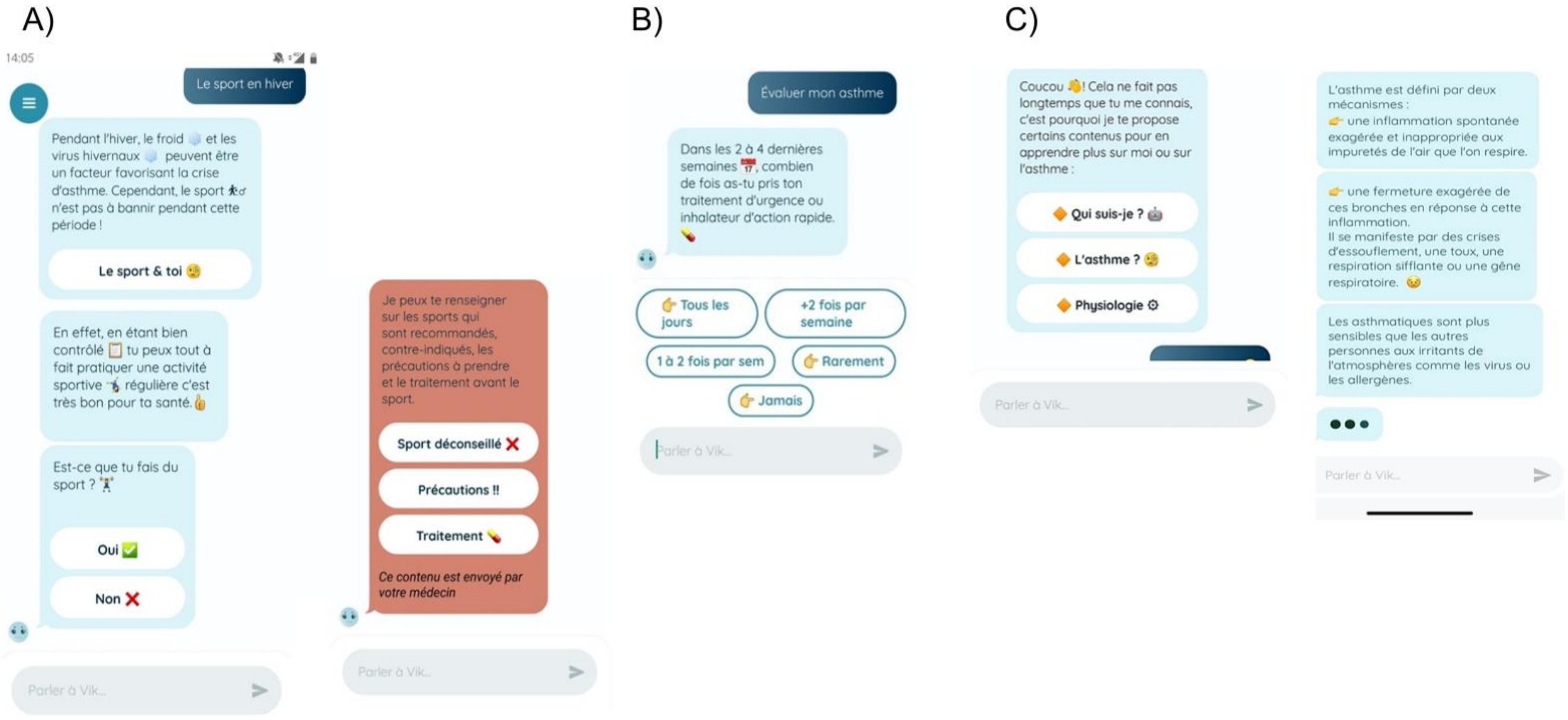

This figure shows three examples (A, B, and C) of screenshots of the application screen seen by the patient. The screenshots show input from the patient, responses from the Chatbot, and the bar where patients input text.

A) In this example, the patient asked about exercising during the winter. This question is related to the domain “physical activity”. The Chatbot responded that, “during winter, the cold and the seasonal viruses can contribute to an increased risk of asthma attacks. However, you should not stop exercising during this period!” The patient then chose to learn more about “sport and you”. The chatbot responded, “Indeed, when your asthma is well controlled, you can absolutely engage in regular physical activity—it's very good for your health. Do you play any sports?” The Chatbot proposes two answers: “yes” or “no”. When the patient does not respond, the Chatbot reinitiates the conversation by saying, “I can give you information about which sports are recommended or not advised, the precautions to take, and the treatment to use before exercising”. The Chatbot proposes the following responses to continue the conversation, “sports that are not recommended”, “precautions”, and “treatments”

B) This example shows how a patient could use the application to evaluate their own symptoms. The patient’s input states “Evaluate my asthma”. The Chatbot responds, “In the last 2-4 weeks, how many times did you take your emergency treatment or your rescue inhaler?” The patient can then choose among the following response options: “Every day, more than twice a week, 1-2 times a week, rarely, never”. The Chabot continues to ask questions, and based on the patients’ responses may or may not recommend that the patient seek medical care.

C) These screen shots show an example of the application initiating a conversation related to one of the three therapeutic education domains chosen by the patient at the beginning of the intervention. In this case, the Chatbot initiates the conversation by saying, “Hey! You haven’t known me for a long time. Would you like to ask a question to get to know me better or to know more about asthma?” The Chatbot then proposes three possible questions, “Who am I” (which would explain how the application works), “asthma”, and “physiology”. In this example the subject chose “asthma”, so the application explains that “asthma is defined by two mechanisms: (1) A spontaneous, exaggerated, and inappropriate response to the impurities in the air that we breathe, (2) an exaggerated narrowing of the bronchi in response to this inflammation. It manifests as episodes of shortness of breath, coughing, wheezing, or breathing discomfort. People with asthma are more sensitive than other people to irritants in the environment, like viruses or allergens”.

Supplemental Figure 2: Workflow of the Chatbot application in asthma healthcare delivery.

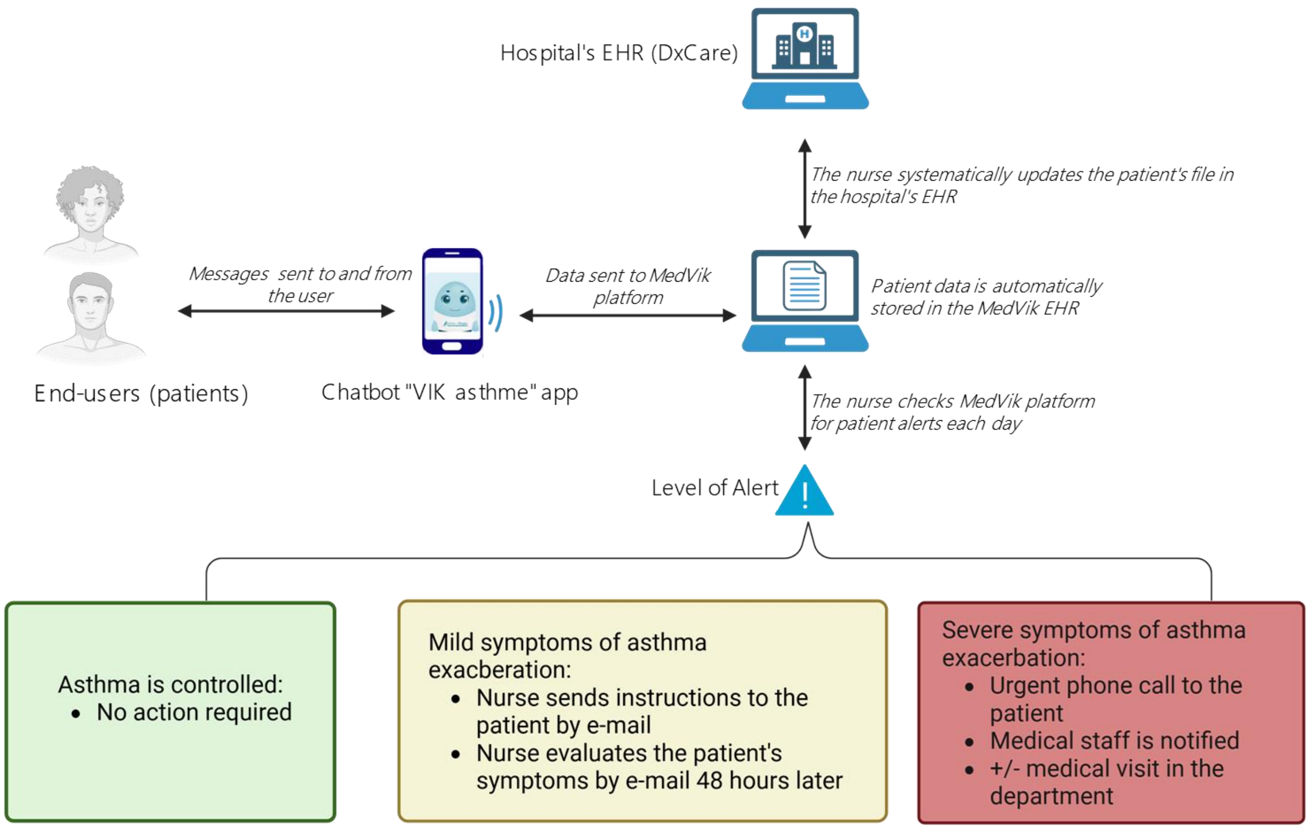

The patients sent messages to the Chatbot, either spontaneously or in response to a Chatbot prompt. Data from the “Vik asthma” app was then automatically sent and stored locally in the MedVik Electronic Health Record (EHR), which was specifically created for the study. Trained nurses checked the MedVik platform each day. They then systematically updated the patient’s file in the Hospital’s EHR (DxCare). The nurse also checked the MedVik platform for alerts. Depending on the words used by the patient, or their input frequency, the application’s algorithm classified alerts as green, orange, or red, which were displayed on a specific dashboard reviewed by nursing staff each morning. An orange-level alert triggered an email contact to the patient to determine whether the patient’s asthma symptoms were worsening, and to help manage a potential episode. A red-level alert resulted in a phone call, and potentially an unscheduled visit to the pulmonology department.

Supplemental Figure 3: Screenshot of the dashboard seen by the nurses on the MedVik platform, with an example of an alert

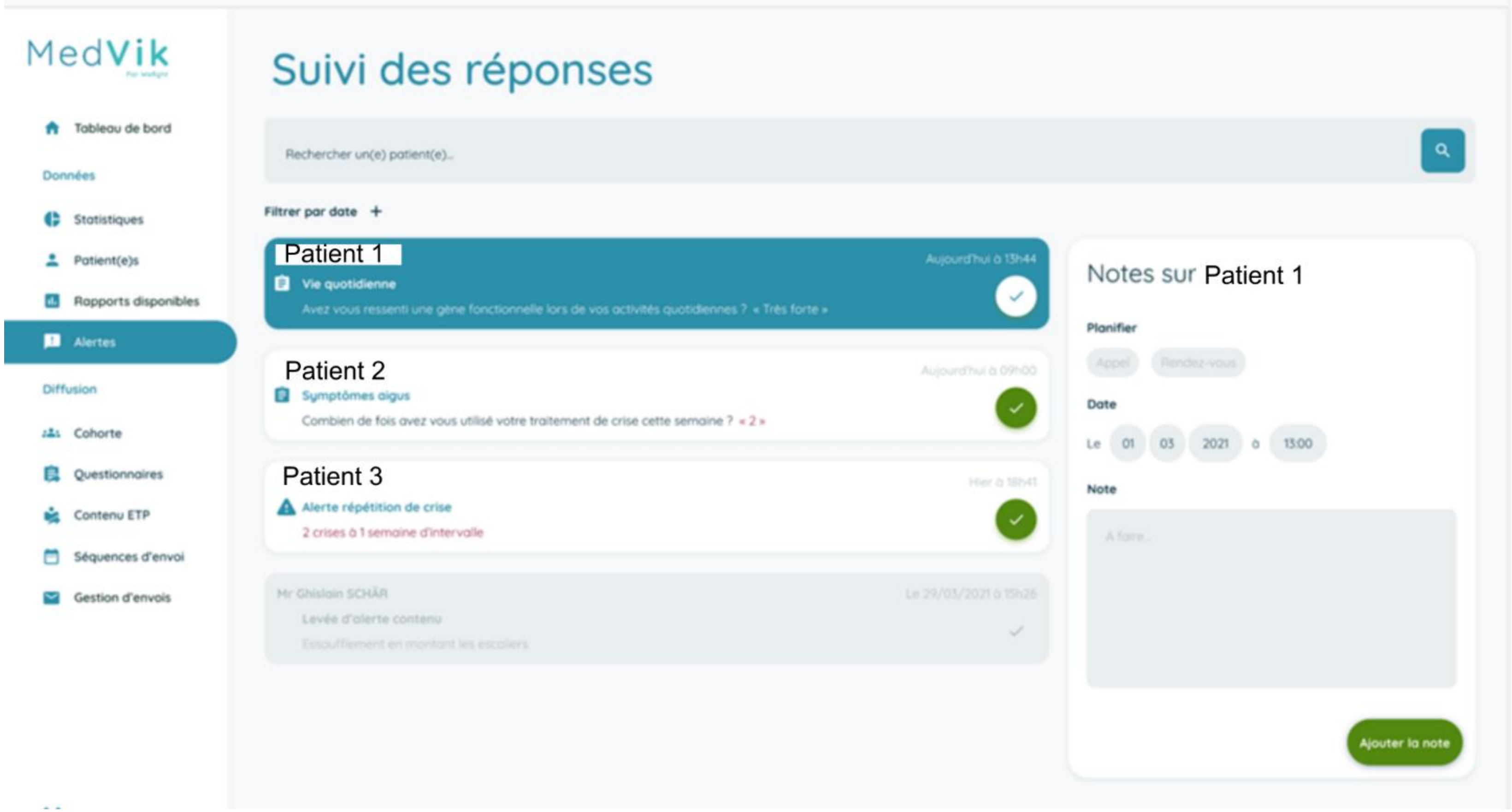

This screenshot of the dashboard from the MedVik platform shows an example of three alerts. For Patient 1, the alert was sent because the patient responded "[yes], very strong [impairments]" to the question, "have you felt any functional limitations in your daily activities?" The nurse clicked on the alert notification, and has the option of sending a note via email, scheduling a phone call, or scheduling a visit. For Patient 2, the application had asked, "How many times have you used your rescue treatments this week", and the patient responded, "2". For Patient 3, the patient had used the phrase, "two asthma exacerbations one week apart" in their exchanges with the Chatbot. For each alert the nurse can either send a note, schedule a phone call, or schedule a visit.

Supplemental Figure 4: The number of connections, defined as the number of times the patient opened and interacted with the Chatbot application on their smartphone between their inclusion in the study and the end of the study

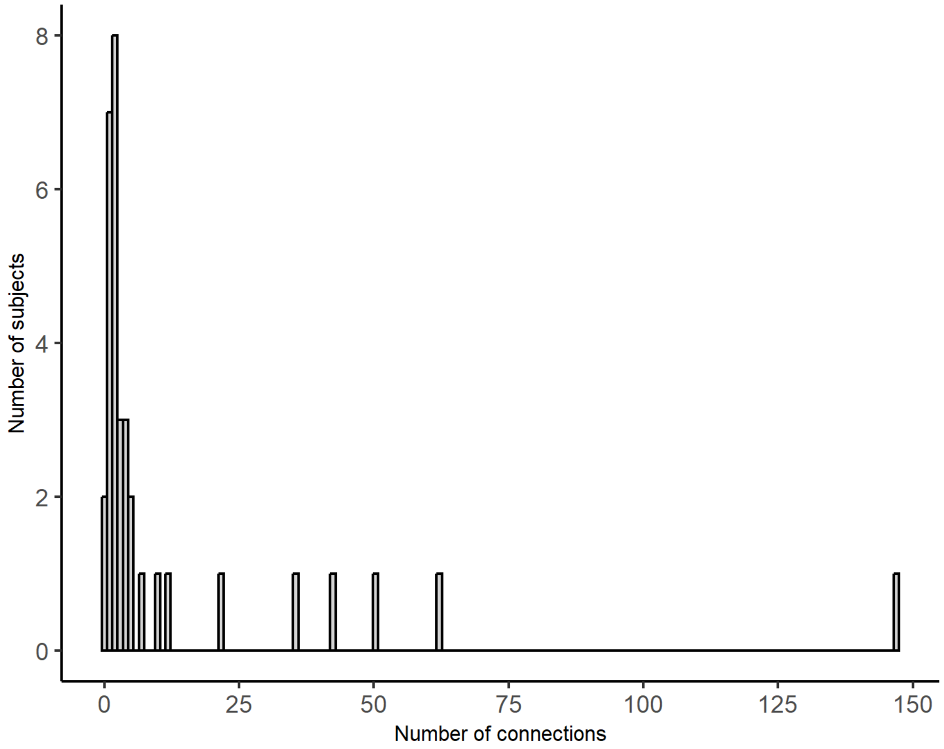

Supplement: Supplementary file 1 [file 00361-2025.SUPPLEMENT.pdf]
